# ARHGAP15 promotes metastatic colonization in gastric cancer by suppressing RAC1-ROS pathway

**DOI:** 10.1371/journal.pgen.1010640

**Published:** 2023-02-21

**Authors:** Fei-fei Zhang, Chen Jiang, Dong-ping Jiang, Yu-zhu Cui, Xin-yue Wang, Liang-zhan Sun, Miao Chen, Ka-On Lam, Sha-yi Wu, Krista Verhoeft, Dora Lai-wan Kwong, Xin-Yuan Guan

**Affiliations:** 1 Department of Clinical Oncology, Li Ka Shing Faculty of Medicine, The University of Hong Kong, Hong Kong, China; 2 Department of Pathology, Sun Yat-sen University Cancer Center, Guangzhou, China; 3 State Key Laboratory of Oncology in Southern China, Sun Yat-sen University Cancer Center, Guangzhou, China; 4 Advanced Energy Science and Technology Guangdong Laboratory, Huizhou, China; 5 MOE Key Laboratory of Tumor Molecular Biology, Jinan University, Guangzhou, China; St Jude Children’s Research Hospital, UNITED STATES

## Abstract

The molecular mechanism of tumor metastasis, especially how metastatic tumor cells colonize in a distant site, remains poorly understood. Here we reported that ARHGAP15, a Rho GTPase activating protein, enhanced gastric cancer (GC) metastatic colonization, which was quite different from its reported role as a tumor suppressor gene in other cancers. It was upregulated in metastatic lymph nodes and significantly associated with a poor prognosis. Ectopic expression of *ARHGAP15* promoted metastatic colonization of gastric cancer cells in murine lungs and lymph nodes *in vivo* or protected cells from oxidative-related death *in vitro*. However, genetic downregulation of *ARHGAP15* had the opposite effect. Mechanistically, ARHGAP15 inactivated RAC1 and then decreased intracellular accumulation of reactive oxygen species (ROS), thus enhancing the antioxidant capacity of colonizing tumor cells under oxidative stress. This phenotype could be phenocopied by inhibition of RAC1 or rescued by the introduction of constitutively active RAC1 into cells. Taken together, these findings suggested a novel role of ARHGAP15 in promoting gastric cancer metastasis by quenching ROS through inhibiting RAC1 and its potential value for prognosis estimation and targeted therapy.

## Author summary

Although metastasis is an extremely insufficient process, it constitutes the major cause of gastric cancer related death. Up to present, little is known about the metastatic colonization, especially how the quite limited tumor cells survive in distant sites to develop metastatic lesions. Here, we reported a Rho GTPase activating protein ARHGAP15, which was identified as a leading upregulated gene in metastatic lymph nodes but previously reported as a tumor suppressor in other malignancies, promoted metastatic colonization of gastric cancer *in vivo* and protected cell from oxidative-related death *in vitro*. Further studies indicated that ARHGAP15 suppressed RAC1 and then decreased intracellular reactive oxygen species produced by NOX2, a downstream gene of RAC1, thus protecting colonizing tumor cells from oxidative stress. Our research contributed to a more comprehensive and detailed understanding of the dynamics of metastatic colonization and more importantly, demonstrated the pro-metastatic role of ARHGAP15 for the first time, providing novel insights into the role of this Rho GTPase activating protein.

## Introduction

Gastric cancer (GC) is the fifth most common cancer and the second leading cause of cancer related death worldwide [1]. GC is considered curable in early stages, but the prognosis is bleak once metastasis occurs [2,3]. Therefore, an improved understanding of molecular mechanism underlying GC metastasis will facilitate developing novel therapeutic approaches to reduce the mortality of GC.

Activating invasion and metastasis is one of the cancer hallmarks [4] and responsible for most cancer related death [5–7]. Generally, metastasis is believed to be a multistage process, including local invasion, intravasation, survival in circulation, extravasation and colonization [8–10]. This is an extremely inefficient process as the majority of metastatic cancer cells would die due to loss of growth signals supported by primary tumor microenvironment and attack from immune system or shear forces of blood stream [9]. As a result, only a quite small number of them survive at a distant site to form micro-metastases [11–13]. Now the molecular strategies employed by the tumor cells to leave primary tumor are being intensively investigated, less clear however is how the metastatic tumor cells survive and colonize in a foreign environment [9].

To address this question, we compared the gene expression profile between the matched primary tumor and metastatic lymph nodes from 8 GC patients. In data analysis, many genes of potential functional importance to metastasis were identified, including *ARHGAP15*, a Rho GTPase activating protein (Rho GAP). Compared with the primary tumor, it was upregulated in lymph nodes from 6 of 8 cases, implying its pro-metastatic role in gastric cancer. Interestingly, *ARHGAP15* has been reported as a tumor suppressor in all published studies, such as lung cancer [14], breast cancer [15], colon cancer [16] and glioma [17], which is contradictory to our findings. Therefore, we proceeded to investigate the exact role of *ARHGAP15* in gastric cancer metastasis to see if it was a “double-edged sword” under different circumstances.

## Results

### *ARHGAP15* was identified as a leading upregulated gene in the metastatic lymph nodes

**Fig 1:**
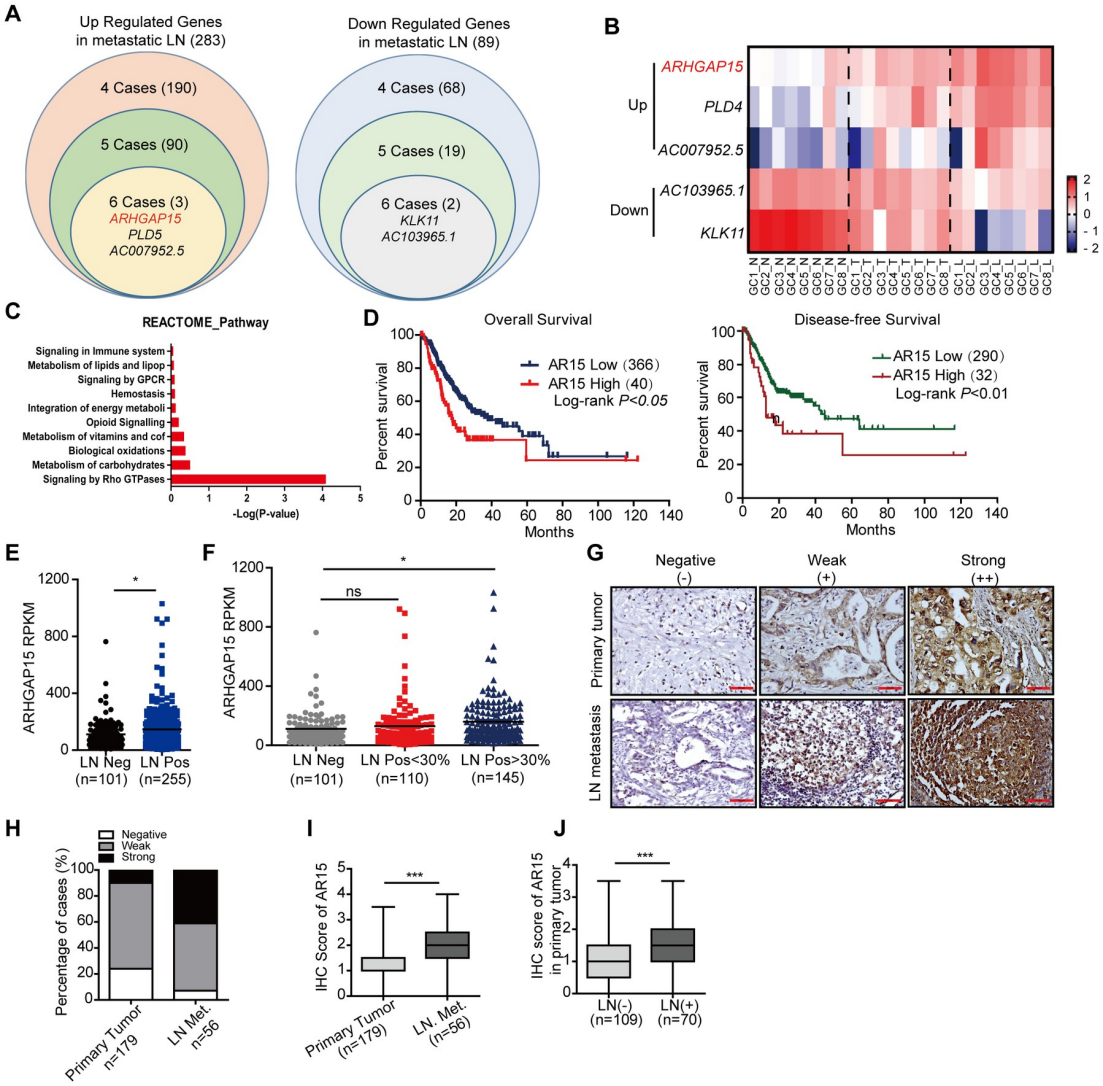
*ARHGAP15* was identified as a leading upregulated gene in the metastatic lymph nodes. **(A)**: Differentially expressed genes in metastatic lymph nodes (LN) were identified. The frequently LN metastatic-dysregulated genes which appeared in at least 6 out of 8 cases were shown. **(B)**: Heatmap showed the 3 leading up-regulated and 2 down-regulated genes in LN Met. **(C)**: GO analysis indicated that dysregulated genes in LN metastasis were enriched in Rho GTPase signaling pathway. **(D)**: Based on TCGA data, *ARHGAP15* expression was associated with unfavorable overall survival and disease-free survival. **(E)**: Based on TCGA data, *ARHGAP15* expression was up-regulated in LN metastasis positive group than the negative group. **(F)**: Comparison of *ARHGAP15* expression between the group without LN metastasis, the group whose metastatic LNs was less than and over 30% of total. **(G-J)**: TMA analysis suggested ARHGAP15 level was upregulated in metastatic lymph nodes. G: The ARHGAP15 negative staining (score 0), weak positive staining (score 0.5–2.5) and strong positive staining (score 3–4) were shown in primary tumor and LN metastasis respectively. H: The histogram showed the proportion of cases with negative staining, weak positive staining and strong positive staining in the primary tumor and LN metastasis respectively. I: ARHGAP15 was significantly up-regulated in the LN metastasis than primary tumor tissue. J: ARHGAP15 was up-regulated in the primary tumor of the cases with LN metastasis. ns: no significance; *, *P*<0.05; **, *P*<0.01; ***, *P*<0.001.

To identify differentially expressed genes with significance between primary GC and lymph node metastasis, RNA sequencing of primary tumor (T), matched metastatic lymph node (LN) and adjacent non-tumor tissue (N) in 8 GC patients was performed. The sequencing data was subsequently analyzed case by case to search for the most frequently and significantly dysregulated genes in the metastatic site. Briefly, metastasis associated up-regulated genes were selected according to the following criteria: gene expression level in metastatic LN was at least 2 folds higher than that of both T and N tissue, meanwhile, gene expression was also higher in the T than in the N. For the metastasis associated down-regulated genes, the selection criteria were listed as follows: gene expression in N should be greater than that in the T and the gene expression in the LN should be decreased at least 0.5 and 0.8-fold when compared to the N and T tissue, respectively. According to the criteria mentioned above, we obtained a gene list for each case and drew a Venn diagram for all 8 cases. The metastasis associated genes which appeared in at least 4 out of 8 cases were identified, including 283 up-regulated and 89 down-regulated genes (Fig 1A). Further analysis revealed 3 most frequently and significantly upregulated and 2 downregulated genes in 6/8 LN metastatic cases (Fig 1A and 1B). In addition, Rho GTPase signaling pathway was significantly enriched in GO analysis of the dysregulated genes (Fig 1C). Meanwhile, *ARHGAP15*, a Rho signaling regulator, was identified as one of the leading genes that was significantly upregulated in the LN metastases (Fig 1B). Considering Rho GTPase signaling pathway was enriched in the GO analysis, we focused on this gene to uncover its significance in gastric cancer metastasis.

### Association of *ARHGAP15* expression with outcomes of GC patients

To further study the association of *ARHGAP15* expression and GC patients’ outcomes, the human GC *ARHGAP15* gene expression and clinical data from the TCGA database were analyzed. High expression of *ARHGAP15* was found to be significantly related with a reduced overall survival and disease-free survival (Fig 1D). Furthermore, the GC patients were divided into LN metastasis positive and negative groups. As expected, LN metastasis was found to be significantly associated with the outcomes of GC patients (S1A Fig). Next, the *ARHGAP15* expression was assessed in these two groups and it was revealed that *ARHGAP15* was highly expressed in the primary site of GC patients with LN metastases (Fig 1E). According to the ratio of the positive metastatic lymph nodes to all the lymph nodes examined, the LN metastasis positive group was further subdivided into groups with more than 30% and less than 30% positive lymph nodes. Similarly, higher proportion of metastatic lymph nodes predicted shorter survival, while there was no significant difference in survival between the group without lymph node metastasis and the group with less than 30% metastatic lymph nodes (S1B Fig). In addition, the higher proportion of metastatic lymph nodes, the higher expression of *ARHGAP15* in the primary site of GC patients (Fig 1F).

As the above TCGA data only described the expression profile of the primary tumor, analysis based on tissue microarray (TMA) was performed with immunohistochemistry (IHC) staining to assess the level of ARHGAP15 in metastatic LN. The tissues were scored from 0–4 and were divided into strongly positive (score 3–4), weak positive (score 0.5–2.5) and negative (score 0) expression groups (Fig 1G). The cohort of the TMA database included 179 primary GC tissues and 56 metastatic LNs (Fig 1H). Being consistent with the TCGA data, *ARHGAP15* expression was upregulated in the metastatic LN when compared with that in the primary site (Fig 1H and 1I). Furthermore, it was also demonstrated that *ARHGAP15* expression in the primary tumor tissue was significantly upregulated in the GC patients with LN metastasis compared with those without LN metastasis (Fig 1J). These findings indicated that *ARHGAP15* was significantly associated with a more advanced disease.

### ARHGAP15 enhances metastatic colonization *in vivo*

**Fig 2:**
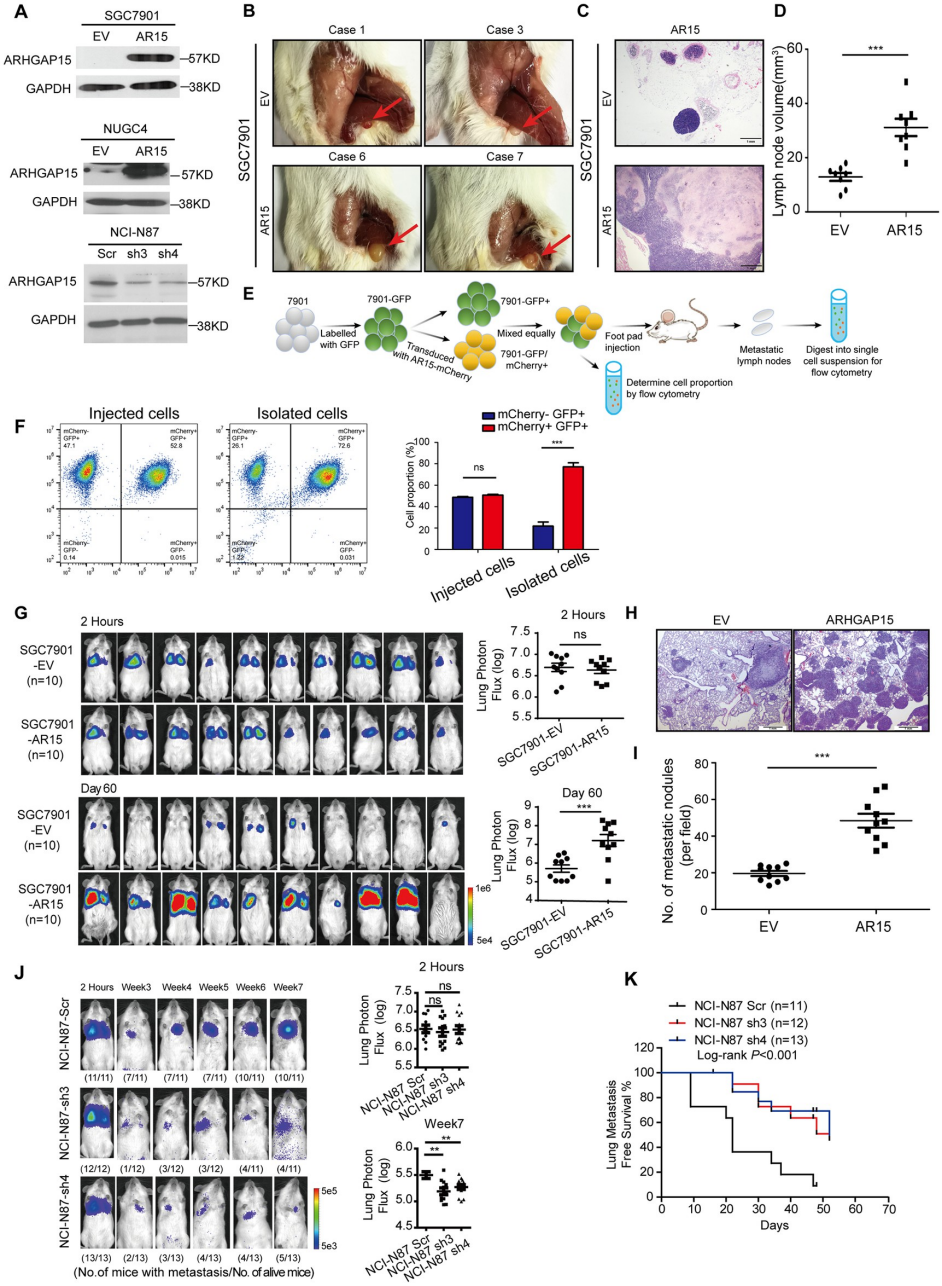
ARHGAP15 enhanced lung metastatic colonization *in vivo*. **(A)**: ARHGAP15 expression level was determined by western blot. **(B-D)**: Spontaneous lymph node metastasis model was established by injection of GC cells into the footpad of mice. 30days after injection, mice were sacrificed to isolate lymph nodes for gross picture taking (B); H&E staining (C) and comparison of lymph node volume (D). **(E)**: Schematic of experiment design for analyzing cell proportion to explain the mechanism of *ARHGAP15* upregulation in metastatic lymph nodes. **(F)**: The proportion of GFP+ and GFP+/mCherry+ GC cells was determined by flow cytometry with cells collected before footpad injection or isolated from lymph nodes. **(G)**: SGC7901 cells with or without *ARHGAP15* overexpression were injected into the tail vein of mice for lung metastatic colonization analysis (n = 10 per group). Bioluminescent imaging quantification and images were taken at 2 hours and day 60 post injection. **(H-I)**: Pulmonary metastasis was shown by H&E staining (H) and the number of which was counted for comparison (I). **(J)**: NCI-N87 cells with or without *ARHGAP15* silencing were intravenously injected for lung colonization analysis. Shown were bioluminescent imaging for the annotated time points post injection. **(K)**: The metastatic status was weekly monitored by in vivo imaging and the time points that signal was detected for the first time after the disappearance of initial signal were documented for lung metastasis free survival analysis. Data was shown as mean±SD. ns: no significance, ** *P*<0.01. *** *P*<0.001.

To determine whether ARHGAP15 is a functional regulator of metastatic colonization ability, it was stably overexpressed in GC cell lines SGC7901, NUGC4 and knocked down in GC cell line NCI-N87. The overexpression and knock down was confirmed by Western Blot (Fig 2A). Then we examined metastatic potential of *ARHGAP15* expressing GC cells in lymph nodes by foot pad injection model. The results indicated that ARHGAP15 promoted GC cell aggressiveness as larger lymph nodes filled with metastatic cells was observed (Fig 2B, 2C and 2D). Furthermore, we found that GC cells with ectopic expression of *ARHGAP15* were enriched in metastatic lymph nodes, which might explain the higher abundance of ARHGAP15 in metastatic lymph nodes than the primary tumor (Fig 2E and 2F). In the tail-vein injection model, the GC cells were labelled by luciferase before injection, which would allow us to monitor the metastasis by bioluminescent imaging. The results of *in vivo* imaging suggested that *ARHGAP15* overexpression significantly increased metastatic colonization in murine lungs after intravenous injection of SGC-7901 and NUGC4 cells (Fig 2G and S2 Fig). Meanwhile, pulmonary metastasis was histologically confirmed by H&E staining (Fig 2H) and the number of metastatic nodules in murine lungs from different groups was also compared (Fig 2I). In addition, *ARHGAP15* was silenced in NCI-N87 cells by two distinct shRNAs, which significantly retarded metastatic colonization at lungs (Fig 2J) and extended the lung metastatic-free survival of the mice (Fig 2K).

### ARHGAP15 promotes ECM-detached tumor cell survival *in vivo* and *in vitro*

Metastatic colonization is a very inefficient event and quite few metastatic tumor cells can successfully colonize at distant sites. In this study, the metastatic colonization capability of GC cells was evaluated with the above-mentioned lung-metastatic mouse model by intravenous injection with one million cells. The results indicated that the injected cells stayed in the lungs at 30 minutes after injection (S3A Fig). The bioluminescent signal of tumor cells peaked at 6 hours, began to decrease at 16 hours, and finally disappeared at 72 hours after cell injection (S3B Fig). The serial observation of surviving tumor cells in lungs demonstrated that quite limited GC cells could survive in the lungs.

**Fig 3:**
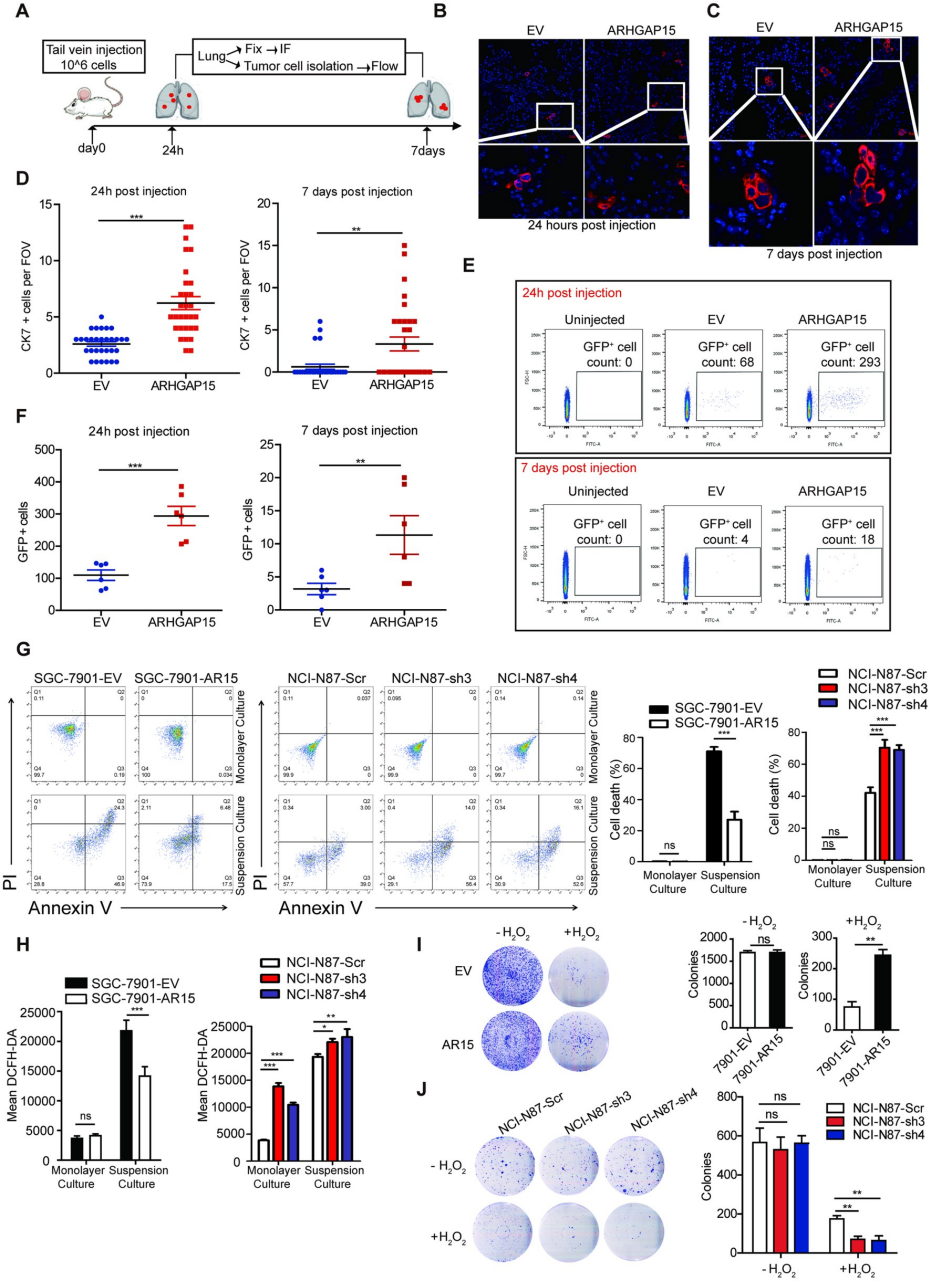
ARHGAP15 promotes ECM-detached tumor cell survival and colony formation under oxidative stress. **(A)**: Schematic representation of studying gastric tumor cell survival *in vivo*. 10^6^ cells were injected and then the number of the survived tumor cells in the lung was quantified at indicated time points. **(B-D)**: The tumor cells were recognized by anti-CK7 antibody in immunofluorescent staining. Survived tumor cells in lungs were quantified at 24 hours (**B and D**) and 7 days (**C and D**) post injection. 3 mice per group and tumor cells from 30 field of views (FOV) for each group were counted. **(E-F)**: The injected tumor cells were marked by GFP and isolated from murine lungs at 24h or 7days post injection, then they were quantified by flow cytometry. The number of the GFP^+^ cell out of 10^6^ lung cells was quantified. 6 mice per group at each time point. **(G)**: GC cells with overexpressed or silenced *ARHGAP15* were cultured in suspension or monolayer condition and the cell death rate of which was determined by flow cytometry with Annexin V/PI dual staining. **(H)**: The level of intracellular ROS of GC cells under different culture conditions was determined by flow cytometry after DCFH-DA staining. **(I-J)**: The colony formation assays were performed by seeding single cells in 6 well plates for 14 days under the indicated treatment. (I): Colony formation assay for SGC7901 cells with or without *ARHGAP15* overexpression under the treatment H_2_O_2_. (J): Colony formation assay for NCI-N87 cells with or without *ARHGAP15* knockdown under the treatment of H_2_O_2_. Data was shown as mean±SD. ns: no significance, *P<0.05, **P<0.01, ***P<0.001.

To explore the effect of ARHGAP15 on tumor cell survival and colonization in lungs, one million *ARHGAP15*-transfected or vector-transfected GC cells were injected through the murine tail vein. Then the mice were sacrificed after 24 hours or 7 days post injection. Survived tumor cells were detected by immunofluorescent (IF) staining or flow cytometry assay (Fig 3A). In IF staining, the tumor cells were recognized by human specific cytokeratin7 (CK7) antibody, the result of which showed that mice in *ARHGAP15*-transfected group had increased number of survived GC cells in lungs than that in control group at both 24hours and 7days post injection (Fig 3B, 3C and 3D), implying that ARHGAP15 played an important role in tumor metastatic colonization. In addition, the tumor cells were labelled with GFP prior to tail vein injection so that they could be quantified by flow cytometry, the result of which also suggested that *ARHGAP15* overexpression enhanced early seeding and survival of GC cells in murine lungs (Fig 3E and 3F). Interestingly, we also noticed that the survived GC cells in the lungs dramatically decreased at day 7 when compared with those at 24 hours (Fig 3F). Finally, we attempted to mimic the harsh conditions under which the circulating tumor cells were by suspension culture *in vitro*. As shown by flow cytometry, tumor cell viability was not significantly affected by genetic upregulation or downregulation of *ARHGAP15* under classical monolayer culture conditions, but the situation under suspension culture conditions was quite the opposite: ectopic expression of *ARHGAP15* protected tumor cells from death whereas knockdown of it enhanced cellular death (Fig 3G). As accumulating evidence showed that the strikingly increased intracellular reactive oxygen species (ROS) caused by detachment from extracellular matrix (ECM) greatly contributed to the anoikis of metastatic cancer cell [18–20], we proceeded to determine the intracellular ROS level of GC cells with *ARHGAP15* overexpression or knock-down under monolayer or suspension culture conditions by flow cytometry. As anticipated, overexpression or knock-down of *ARHGAP15* attenuated or exacerbated the sharp increase of intracellular ROS for cells cultured in suspension respectively. Under monolayer culture conditions, a mild rise of intracellular ROS was also observed in cells with knock-down of *ARHGAP15* but not in those with overexpression (Fig 3H). Collectively, these data implied the protective effect of ARHGAP15 on circulating tumor cells might be related with regulation of ROS.

### ARHGAP15 promotes GC cell colony formation under oxidative stress

Once the metastatic tumor cells successfully completed extravasation, the following colonization step is critical for a single cancer cell to develop metastasis. Therefore, a foci formation assay was performed to evaluate the *in vitro* colony formation capability of *ARHGAP15* overexpressed or silenced cells treated with or without H_2_O_2_, respectively. After two weeks, no significant difference in the number of colonies was observed between *ARHGAP15* overexpressing cells and control cells if cells were cultured without H_2_O_2_ (Fig 3I). However, under oxidative stress, colony formation capability of the control cells was impaired to a lager extent than *ARHGAP15* overexpressing cells. (Fig 3I). As expected, the number of colonies was significantly decreased in *ARHGAP15* silenced cells under culture conditions with H_2_O_2_, but not under traditional culture conditions. (Fig 3J).

### ARHGAP15 protects GC cells from oxidative stress-induced cell death

**Fig 4:**
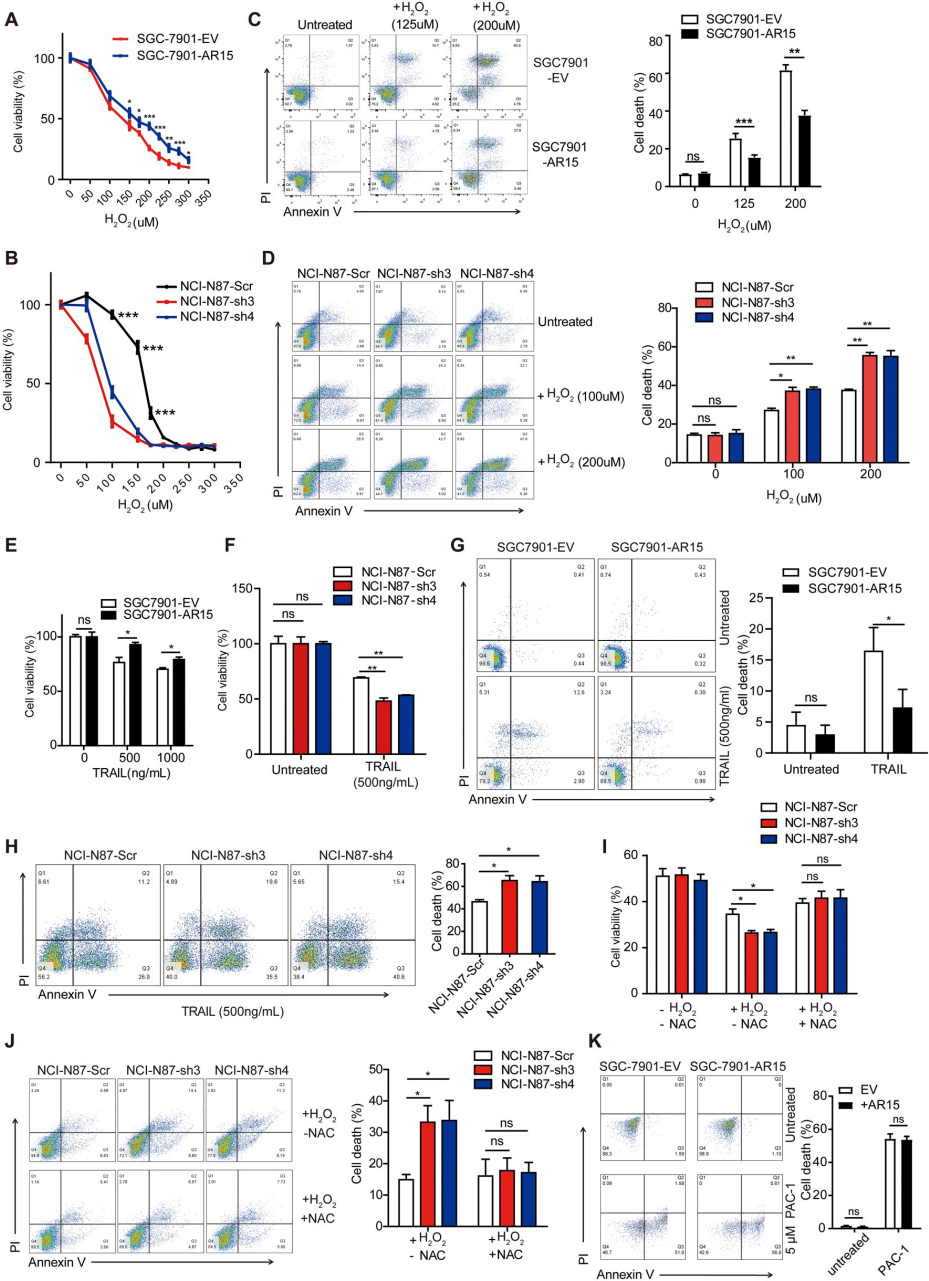
ARHGAP15 protects GC cells from oxidative stress-induced cell death. The cell viability of GC cells with overexpressed *ARHGAP15* (**A**) or silenced *ARHGAP15* (**B**) under different concentration of H_2_O_2_ was determined by XTT assay. **(C-D)**: The proportion of dead cells was determined by flow cytometry after dual staining of Annexin V and PI. (C): The cell death status of SGC7901 cells with or without *ARHGAP15* overexpression under the treatment of indicated H_2_O_2_ concentration. (D): The cell death status of NCI-N87 cells with or without silenced *ARHGAP15* under the treatment of indicated H_2_O_2_ concentration. **(E-F)**: The GC cell viability after TRAIL treatment was evaluated by XTT assay. (E): The fraction of survived SGC7901 cells with or without *ARHGAP15* overexpression upon treatment of TRAIL. (F): The fraction of survived NCI-N87 cells with or without silenced *ARHGAP15* upon treatment of TRAIL. **(G)**: The cell death status of SGC7901 cells with or without *ARHGAP15* overexpression under treatment of TRAIL. **H**: The cell death status of NCI-N87 cells with or without *ARHGAP15* knockdown under treatment of TRAIL. **(I-J)**: The recovery effect of NAC treatment (500mM) on survival of NCI-N87 cells with *ARHGAP15* knockdown was revealed by XTT (I) and flow cytometry assay (J). **K**: The cell death rate of SGC7901 with or without overexpression of *ARHGAP15* under treatment of PAC-1, a procaspase-3 activator. Data was shown as mean±SD. ns: no significance, *P<0.05, **P<0.01, ***P<0.001.

To further explore the effect of oxidative stress on GC cell survival, XTT assay was performed to evaluate cell viability under different levels of oxidative stress for both *ARHGAP15* overexpression and knockdown cells. The results showed that cells with overexpression of *ARHGAP15* had better viability than the control cells (Fig 4A), while cells with knock-down of *ARHGAP15* survived fewer than those transduced with scramble shRNA after H_2_O_2_ treatment (Fig 4B). Next, the H_2_O_2_-induced cell death was also quantified by Annexin V and PI staining, the result of which showed that ARHGAP15 significantly reduced H_2_O_2_-induced cell death (Fig 4C) and the cells tended to die if *ARHGAP15* was silenced (Fig 4D).

TNF-related apoptosis-inducing ligand (TRAIL) is conditionally secreted by immune cells such as natural killer (NK) cells and serves as a pro-apoptotic effector in the immune surveillance of cancer through inducing tumor cell intracellular ROS generation [21,22]. Therefore, we investigated the GC cell survival under the TRAIL-induced oxidative stress by XTT assay. The results indicated that ARHGAP15 promoted cell viability under TRAIL treatment (Fig 4E). On the contrary, silencing *ARHGAP15* decreased the cell viability significantly (Fig 4F). These phenotypes were later confirmed by flow cytometry analysis (Fig 4G and 4H). All these *in vitro* assays suggested the pivotal role of ARHGAP15 for cell survival under oxidative stress.

Next, we studied if the anti-oxidative stress function of ARHGAP15 was through ROS regulation. When intracellular ROS was eliminated by a ROS scavenger NAC (N-Acetyl Cysteine), the phenotype induced by downregulation of *ARHGAP15* was rescued, as shown in XTT assay (Fig 4I) and flow cytometry assay (Fig 4J). To further exclude the possibility that the protective role of ARHGAP15 depended on preventing cells from apoptosis directly rather than oxidative stress induced death, we proceeded to determine whether other pro-apoptosis factors which did not produce ROS still worked upon *ARHGAP15* overexpression. PAC-1, a potent procaspase-3 activator was used to initiate apoptosis directly here [23]. As shown in Fig 4K, *ARHGAP15* overexpression could not prevent PAC-1 induced apoptosis, suggesting no direct effect of ARHGAP15 on cell apoptosis.

### ARHGAP15 decreases intracellular ROS through inhibiting RAC1

**Fig 5:**
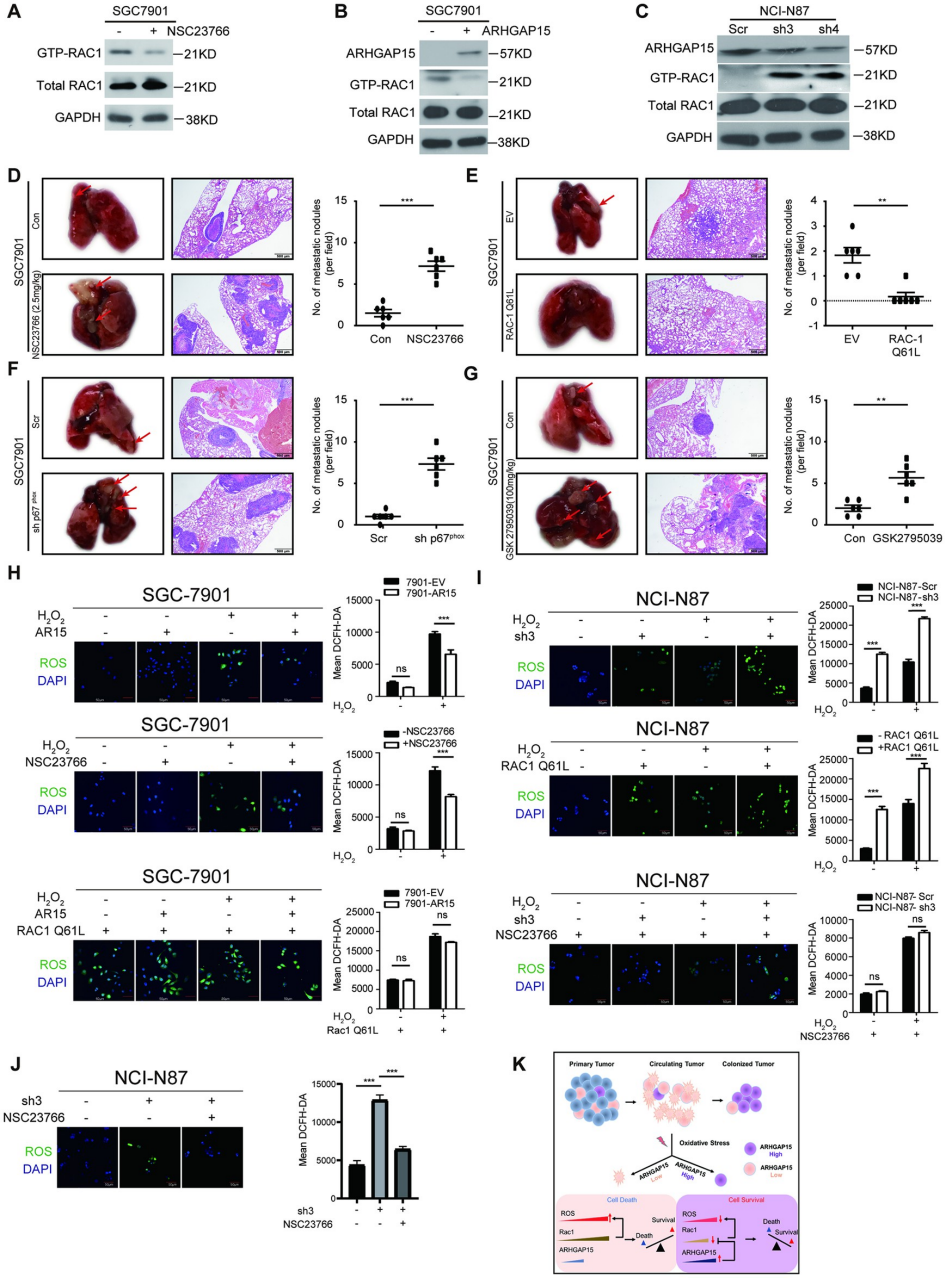
ARHGAP15 decreases intracellular ROS through inhibiting RAC1. **(A)**: Representative western blot images showed the activity of RAC1 was inhibited by NSC23766. GTP-RAC1 was the active form. **(B)**: RAC1 activity was suppressed by *ARHGAP15* overexpression. **(C)**: *ARHGAP15* knockdown enhanced RAC1 activity in NCI-N87 cell. **(D-G)**: *In vivo* metastatic assay based on SGC-7901 cells (n = 6 for each group). Metastatic lesions were shown by gross photos (Left) or H&E slides (Middle). The number of metastatic nodules per field under microscope were counted for comparison (Right). (D): Mice injected with tumor cells through the tail vein were subsequently intraperitoneally injected with corn coil or indicated dosage of NSC23766 every day. (E): Tumor cells stably expressing wild type RAC1 or mutated RAC1 Q61L were injected to form metastasis. (F): Tumor cells with or without silencing p67^phox^ (core subunit of NOX2) were injected. (G): Mice upon tail veil injection of tumor cells were administered with corn oil or indicated dosage of GSK2795039 by intraperitoneal injection every day. **(H-J)**: The intracellular level of ROS of SGC7901 cells (H) and NCI-N87 cells (I and J) under indicated conditions was shown by fluorescent microscope (Left) and determined by flow cytometry (Right) with DCFH-DA staining. AR15: ARHGAP15; NSC23766: RAC1 inhibitor, 20μM; RAC1 Q61L, constitutively active mutant of RAC1. **(K)**: Schematic diagram of ARHGAP15-mediated cell survival under oxidative stress. Circulating tumor cells were confronted with oxidative stress, which meant the intracellular ROS rapidly accumulated and they would die if the ROS level reached the death threshold. To overcome the harsh conditions, they might take advantage of ARHGAP15 to suppress the activity of RAC1, as a result, the intracellular ROS level was decreased, thus protecting the cells from oxidative stress induced cell death. Data was shown as mean±SD. ns: no significance, ***P<0.001.

To further explore the mechanism of ARHGAP15 in ROS regulation, we searched IntAct online database for its potential interactive partner [24]. Among the 6 proteins (RAC1, PRNP, DHPS, FGD2, KDM1A and LRRK2) reported to have possible interaction with ARHGAP15, RAC1 ranked 1^st^ in the MI score, which suggested it was most likely to be the molecular partner of ARHGAP15. To further investigate whether ARHGAP15 regulates the activity of RAC1 in GC cells, the RAC1 activation assay by pulling down the GTP-bound RAC1 was performed. Firstly, the reliability of RAC1 activation assay was tested by treating GC cells with a RAC1 inhibitor (NSC23766), and the result demonstrated that the RAC1 inhibitor could significantly reduce the GTP-bounding RAC1 (Fig 5A). The activity assay was then performed to compare the active RAC1 levels between *ARHGAP15*-transfected and vector-transfected GC cells. The result indicated that *ARHGAP15* overexpression suppressed the RAC1 activity (Fig 5B). Consistently, knockdown of *ARHGAP15* enhanced the activity of RAC1 in both shRNA isoforms (Fig 5C), indicating that ARHGAP15 negatively regulated RAC1 activity. To determine whether the abundance of RAC1 was associated with ARHGAP15, we continued to analyze the association on both transcriptional and protein levels. Based on TMA slides, no significant correlation between the protein level of RAC1 and ARHGAP15 was observed (Spearman ρ = -0.030, *P* value = 0.689). Consistently, quite weak correlation (Pearson’s r = -0.13, *P*<0.01) can be observed at transcriptional level, as shown by TCGA data downloaded from cBioportal [25] (S4 Fig). Taken together, it was the activity, rather than the abundance of RAC1 was regulated by ARHGAP15.

However, whether ARHGAP15 was responsible for the inhibition of RAC1 activity and consequently contributed to promote metastasis *in vivo* remained unknown. To explore this, RAC1 was inhibited by its specific inhibitor NSC23766 or activated by introduction of its constitutively active form, RAC1 Q61L. The data from tail vein injection model suggested that RAC1 suppression and activation respectively promoted (Fig 5D) or demoted (Fig 5E) metastatic colonization in murine lungs. RAC1, a small GTPase which was mainly involved in cytoskeleton reconstruction and cell motility, was also reported to indirectly regulate the production of ROS by integrating with p67^phox^, a subunit of NADPH oxidases (NOX2) [26,27]. Additionally, NOX2 was also reported as the most abundant member of NOX family in gastric cancer [28]. To determine whether the metastatic phenotype mentioned above was NOX2 dependent, we injected SGC-7901 cells with knock down of p67^phox^ into the tail vein. The results showed that silencing p67^phox^ significantly promoted metastasis in *vivo* (Fig 5F). Similar phenotype was also observed when SGC-7901 wild type cells were injected and GSK2795039 [29], a specific inhibitor for NOX2, was administered via intraperitoneal injection every day at a dosage of 100mg/kg (Fig 5G). To further investigate whether ARHGAP15 decreased intracellular ROS through suppressing RAC1, we determined the intracellular level of ROS under different conditions by fluorescent microscope and flow cytometry with DCFH-DA dye. As expected, ectopic expression of *ARHGAP15* diminished intracellular ROS under H_2_O_2_ induced oxidative stress but not traditional culture conditions and this phenotype could be phenocopied by treatment with NSC23766. Interestingly, the intracellular ROS sharply increased if the constitutively active form of *RAC1* (*RAC1 Q61L*) was transfected into the cells, no matter whether *ARHGAP15* was overexpressed or H_2_O_2_ was administered (Fig 5H). However, GC cells experienced a rapid accumulation of intracellular ROS if *ARHGAP15* was silenced, even under environment without H_2_O_2_ induced oxidative stress. This phenotype could be phenocopied by transfection of *RAC1 Q61L* and rescued by NSC23766 treatment (Fig 5I). Additionally, the increased intracellular ROS induced by silencing *ARHGAP15* was rescued by NSC23766 treatment (Fig 5J). Collectively, these findings demonstrated ARHGAP15 protected cells from excessive accumulation of ROS via inhibiting RAC1 activity.

## Discussion

Metastasis is the main cause of GC-related death. In our recent attempt to identify GC metastasis associated genes, RNA-seq was performed to compare gene expression profiles between primary tumor and the matched metastatic lymph nodes. Here, *ARHGAP15* was identified to be significantly upregulated in the LN metastasis and high *ARHGAP15* expression was associated with a more advanced disease. With the functional study *in vivo* and *in vitro*, we demonstrated that ARHGAP15 could promote gastric cancer metastatic colonization.

Once the tumor cells disseminated from the primary tumor, the majority of them undergo cell death on the way, leading to extremely low efficiency of metastatic colonization [30]. Oxidative stress encountered during metastasis is one of the major causes of cell death. To withstand that, the metastatic tumor cells have to turn on an effective antioxidative system to limit accumulation of the intracellular ROS. In this study, we found ARHGAP15 inhibited the activity of RAC1 to alleviate intracellular ROS accumulation, thus protecting tumor cells from oxidative stress induced cell death (Fig 5K). Additionally, the colony formation ability of single tumor cell was critical to develop metastasis in a secondary site and we demonstrated that upregulation of *ARHGAP15* enhanced the colony formation capability of single tumor cell under oxidative stress. These findings suggested that the successfully seeded metastatic tumor cells took advantage of ARHGAP15 to cope with oxidative stress and achieved the ability to colonize at distant sites, providing a mechanistic basis for how the metastatic tumor cells overcome the oxidative stress encountered on the way as well as a novel role of ARHGAP15 in this process.

Given that the sustained rise of ROS contributes to genomic instability and drives pro-tumorigenic pathways, antioxidants are supposed to prevent or treat cancers [31,32]. Despite the therapeutic effect of antioxidants were evaluated in many large-scale clinical trials, there were no clear results indicating that cancer progression could be halted by the treatment [33,34]. In contrast, the usage of antioxidant often appeared to increase cancer incidence [35,36]. Piskounova et al. found that oxidative stress decreased distant metastasis and antioxidants exacerbated melanoma progression by promoting metastasis [37]. Recent report also showed that antioxidants supplementation promoted KRAS-driven lung cancer metastasis [38]. In this study, our findings indicated that the metastatic tumor cells with enhanced antioxidant capacity were more likely to survive in the metastatic process and successfully colonize in a distant site. This suggested that antioxidants treatment might promote the survival of metastatic tumor cells to facilitate metastasis, raising the possibility that rather than treating cancer patients with antioxidants, they should be treated with pro-oxidant instead to eradicate the colonized tumor cells.

The ARHGAP15 is a Rho GTPase activating protein (Rho GAP) [39,40]. The Rho GAPs greatly boost the activity of Rho GTPase to hydrolyze GTP, thus “turn off” this molecular switch [41,42]. Conventionally, the Rho GTPases, such as RAS, are supposed to be oncogenes, and the Rho GAPs, such as NF1, are thought to have tumor suppressive roles [43–45]. Here, ARHGAP15, a Rho GAP, was believed to play a tumor suppressive role. Indeed, all previously published studies supported the notion by demonstrating that ARHGAP15 functioned as a tumor suppressor in lung cancer [14], breast cancer [15], colon cancer [16] and glioma [17]. However, our recent findings suggested *ARHGAP15* as a pro-metastasis gene in gastric cancer, which was inconsistent with its phenotype shown in previous studies. As our *in vitro* data showed that *ARHGAP15* overexpression had no effect on GC cell migration and invasion (S5 Fig), the pro-metastatic role of ARHGAP15 might not be through the regulation of cell movement. Therefore, we continued to explore the inconsistency to elucidate its underlying mechanism. In mechanistic study, ARHGAP15 could inhibit RAC1, a well-known Rho GTPase playing critical roles in cell movement [46,47], to decrease intracellular ROS via a non-canonical pathway, thus protecting metastatic GC cells from oxidative stress related death. This may help explain the contradictory role of ARHGAP15 in cancer metastasis under different circumstances. Specifically, during the dissemination, the tumor cells might activate RAC1 to acquire enhanced invasive and migrative abilities to leave the primary tumor [48,49], although at a expense of intracellular ROS accumulation. However, once the tumor cells step into the circulation, they no longer require increased invasion and migration abilities, instead they need to “turn off” RAC1 to reduce ROS production in order to survive at foreign sites [10,50]. Therefore, a gene may have opposite effects in different step of metastasis and its exact role should be determined specifically, rather than being arbitrarily defined as an oncogene or tumor suppressive gene.

In conclusion, we demonstrated ARHGAP15 promoted metastatic colonization by enhancing the antioxidant capacity of gastric cancer cells in a RAC1 dependent way. This study deciphered the underlying mechanism between metastasis and oxidative stress, and it opened a potential new avenue for cancer treatment by targeting ARHGAP15 or its downstream signaling with pro-oxidant in the future.

## Materials and methods

### Ethics approval and consent to participate

This study involving human data and tissue was approved by the Committees for Ethical Review of Research of Xinyang Hospital and written informed consent in accordance with the Declaration of Helsinki was obtained from each patient. The animal experiments were approved from the Committee on the Use of Live Animals in Teaching and Research (CULATR) of the University of Hong Kong (HKU) with an accession number of 3962–16.

### Cell culture

Three human gastric cancer cell lines were used in the study, SGC7901, NUGC4 and NCI-N87. SGC7901 and NUGC4 were derived from lymph node metastatic site, while NCI-N87 was derived from liver metastatic site. All the gastric cancer cell lines were obtained from cell bank, Chinese academy of Sciences, Shanghai. The cells were cultured in complete RPMI1640 medium (Gibco) with 10% fetal bovine serum (FBS) (Gibco) in a humidified 5% CO2 incubator at 37°C. For suspension culture, the conventional culture plate was replaced by 24-well ultralow attachment culture plate (Corning) and 3000 cells were seeded to each well for culture. The 293FT cell line for lentivirus production was purchased from Invitrogen. The 293FT cell was cultured in complete DMEM (high glucose) medium with 10% FBS, 1 mM MEM Sodium Pyruvate (Gibco), 0.1mM MEM Non-Essential Amino Acids (NEAA) (Gibco) and 500μg/ml Geneticin (Gibco) for cell growth and maintenance.

### Human gastric cancer samples

All the clinical samples of gastric cancer patients were collected from Xinyang people’s hospital (Henan). Informed consent was obtained from all patients before the experiment started and the study had been approved by the Committees for Ethical Review of Research of Xinyang people’s hospital. The samples for RNA sequencing were snap-frozen in liquid nitrogen immediately after surgical resection. The samples to construct the tissue microarray (TMA) were fixed in 10% formalin for the following paraffin embedding.

### Animal studies

All animals were used under the license to conduct experiments from Department of Health, Hong Kong. The animal experiments were performed at the Laboratory Animal Unit (LAU) with the approval from the Committee on the Use of Live Animals in Teaching and Research (CULATR No.3962-16) of the University of Hong Kong (HKU). The 4–6 weeks NOD SCID mice were used for tail vein injection model construction, the number of mice used was indicated in figure legends respectively. For the SGC7901 cell, 10^6^ cells were suspended in 200μL RPMI1640 and subsequently injected to each mouse. For the NUGC4 and NCI-N87 cells, 5*10^5^ cells suspended in 200μL RPMI1640 were injected. If needed, 2 hours post cell injection, the bioluminescent imaging was performed to localize the injected cells. Mice were injected with luciferin (150mg/kg) (Goldbio) by intraperitoneal injection and then anesthetized by Ketamine and Xylazine. The photon flux was monitored by the PE IVIS Spectrum in vivo imaging system. The photon signal from the lungs was monitored and quantified by the IVIS software. Compounds were administered by intraperitoneal injection with indicated dosage and frequency if needed. For spontaneous lymph node metastasis model construction, 10^5^ GC cells suspended in 20 μl serum free RPMI1640 medium were inoculated to the left hind footpad of the mouse to generate a primary tumor. 30 days after injection, the mice were sacrificed, and the popliteal lymph nodes from the left hind limbs were isolated for subsequent analysis.

### Transcriptome sequencing (RNA sequencing)

In this study, RNA sequencing was performed with the matched tumor, adjacent non-tumor and metastatic lymph nodes from 8 gastric cancer patients (Annoroad Gene Technology, Beijing). The RNA was isolated by TRIzol (Invitrogen) and then the quality control was performed. Next, enrichment was performed by Oligo dT beads followed by RNA fragmentation, reverse transcription, adapter ligation and amplification. After the library prepared, the sequencing was performed on the Illumina HiSeq (2500). The data analysis consisted of removal of poor-quality reads and sequencing adapters, mapping reads, differential expression gene analysis and identification of related pathways.

### Immunohistochemistry (IHC) staining of tissue microarray (TMA)

The cohort of TMA included 179 gastric cancer specimens and 56 metastatic lymph nodes with formalin fixing and paraffin embedding. Clinicopathological parameters were recorded. The TMA was constructed as described previously [51]. Briefly, the 1mm tissue cylinders in diameter were retrieved from microscopically selected representatives. Then they were transferred into a single recipient TMA block for assembly with a tissue arrayer. 5μm thick consecutive sections of microarray block were made with a microtome and mounted on microscope slides. The TMA slides were pre-warmed in 70°C incubator for 1 hour and followed by deparaffinization and rehydration. Next antigen retrieval was performed with Tris/EDTA pH 9.0 buffer under the 95°C water bath heat for 30 minutes to remove the cross link between formaldehyde and proteins. Then the immunohistochemical staining was completed with a biotin-streptavidin HRP detection kit (OriGene). The experiment followed the instructions: incubating the slides in 0.3% H_2_O_2_ for 15 minutes, washing the slide with TBST for 3 times, blocking in reagent 2 at room temperature for 2 hours, applying the primary antibody (ARHGAP15, 1:100, Thermofisher; RAC1, 1:200, Millipore) overnight at 4°C, rinsing in TBST for 3 times, incubating biotinylated secondary antibody for 1 hour at room temperature, and HRP-linked reagent 4 was applied for 20 minutes at room temperature. For the signaling development, the 3,3’-Diaminobenzidine (DAB) (DAKO) was used and rinsed in the tap water after development. The nucleus was stained with hematoxylin (DAKO). Then the slides were dehydrated, air dried and mounted.

### Western blotting

Western blotting was performed according to the standard protocol. Total cell lysates were made in RIPA-buffer (Thermofisher) containing protease inhibitors cocktail. Protein was transferred to 0.45 μm polyvinylidene fluoride (PVDF) membranes (Millipore). Membranes were blocked with 5% Bovine serum albumin (BSA) (Cell Signaling Technology) for 1 h at room temperature, incubated at 4°C overnight with primary antibody and incubated for 1 h at room temperature with the HRP-conjugated second antibody. Chemiluminescence development and imaging was performed with the Bio Rad ECL reagent. Primary antibody used in this study: ARHGAP15 (1:1000, Thermofisher), RAC1 (1:1000, Millipore), GAPDH (1:2000, Santa Cruz).

### RAC1 pull down assay

The active RAC1 pull down assay was performed with the RAC1 activity assay kit (Millipore) according to instructions. Briefly, the cells were lysed with 1mL cold Mg^2+^ Lysis/Wash buffer (MLB). The lysates were transferred to 1ml tubes on ice. 100μL glutathione agarose was added and the mixture was rocked for 10 minutes at 4°C to preclear the lysate. The agarose beads were collected by spinning for 5 seconds at 14,000g. The supernatant was removed and 500μL per sample was aliquoted on ice for the following steps. For the RAC1 pull down assay, 10 μL of PAK-1 PBD agarose was added per 500ul lysate. The reaction was gently rocked at 4°C for 1 hour. The agarose beads were then collected by spinning for 5 seconds at 14,000g. The supernatant was discarded, and the pellet was washed with MLB for 3 times. The agarose beads were resuspended in 20μL protein loading buffer and denatured at 100°C for 10 minutes. The level of activated RAC1 was then determined by the western blot with RAC1 antibody (Millipore).

### ROS detection

2’,7’-dichlorofluorescin diacetate (DCFH-DA) is a cell-permeable non-fluorescent probe. After diffusion into the cell, DCFH-DA is de-esterified and oxidized by ROS into 2’,7’-dichlorofluorescein (DCF). DCF is highly fluorescent and can be detected by fluorescence spectroscopy (excitation/emission = 488/535). The ROS level in this study was determined by flow cytometry or confocal microscope. Generally, the cells were harvested and suspended as single cells in culture medium. Then the cells were treated with 25μM DCFH-DA (Sigma) for 30min at 37°C in the dark. After staining, the cells were analyzed on flow cytometer. To directly observe the ROS under confocal microscope, the cells were seeded at 5x10^4^ cells/well on 8 well chamber slide (Thermofisher). The cells grew to attach and then the cells were treated with 25μM DCFH-DA (Sigma) for 30min at 37°C in the dark. Then the cells were washed 2 times with PBS and nucleus was stained with DAPI. The signal was observed under the confocal microscope Carl Zeiss LSM 700 directly following 2 times of PBS washing.

### XTT cell viability assay

In the XTT assay, we used the CellTiter AQueous One Solution Reagent (Promega). The experiments were performed according to the instructions. Briefly, 5,000 cells in 100μL medium were seeded into a 96 well flat bottom plate in triplicate. Three control wells containing 100μL of complete growth medium only were set for blank absorbance readings. It usually took 12 hours for the cells to adhere. The medium was changed to RPMI1640 medium without FBS but with different concentration of H_2_O_2_ or TRAIL and incubated for indicated time intervals. Then the medium was changed with fresh RPMI1640 medium and 20μL of CellTiter AQueous One Solution Reagent was added directly to the well. The mixture was incubated for 2 hours and then the absorbance at 490nm was read with a 96 well plate reader.

### Cell death assay by flow cytometry

Cell death rate was determined by flow cytometry with the FITC Annexin V Apoptosis Detection Kit I (BD). The assay was performed as follows: 2x10^5^ cells were seeded in a 6 well plate and cultured for 12 hours. Then the cells were treated with indicated concentration of H_2_O_2_, TRAIL for 24 hours or PAC-1 for 8 hours to induce cell death. All the cells, including those in supernatant, were harvested for further analysis. The cells were washed with cold PBS and resuspended in 100μl binding buffer. The solutions were transferred to a 5ml round bottom tube (Falcon) and the cells were stained with 5μl of FITC Annexin V and 5μl PI. The system was gently mixed and incubated for 15 min at room temperature in the dark. Before running on the flow cytometry, 200μl of PBS was added to each tube. The analysis on flow cytometry was finished within 1 hour.

### Colony formation assay

The colony formation aimed at evaluating cell colony formation capability under oxidative stress. The experiments were performed as described before [52]. Specifically, 5,000 cells for NCI-N87, 4,000 cells for SGC7901 were plated in a 6 well flat bottom plate. The plate was placed in the cell culture incubator with 5% CO_2_ at 37°C for colony formation. It usually took 10–20 days for the gastric cancer cells to form colonies consisting of 50 or more cells. The colonies were fixed with 75% ethanol for 1 hour and then stained with 1 mL 1% (w/v) crystal violet for 1 hour. The plate was scanned, and the number of colonies was counted by the ImageJ.

### Immunofluorescent stain (IF)

The murine lungs with metastatic cells were cut into 5μm sections and mounted on the pre-coated glass slides. The sections were dried at 70°C oven for 1 hour followed by deparaffinization and rehydration. The slides were placed in a slide holder and were washed as follows: xylene 10 minutes for 2 times, 100% ethanol 10 minutes for 2 times, and then running tap water for 1 minute. Then the slides were washed with TBS for 3 times. The retrieval buffer (DAKO) was pre-heated to 95°C by placing the staining jar in a water bath at 95°C. The coverslips were heated at 95°C for 30 min for antigen retrieval. After retrieval, the slides were cooling down at room temperature followed by 3 times washing with TBS. The tissue was blocked with 5% BSA in TBST for 1 hour and incubated with the primary antibody (CK7) (dilution 1:100 in 5% BSA-TBST) in a humidified chamber overnight. The primary antibody was removed, and the slides were washed 3 times in TBS for 5 min each time. From this point onwards, all the steps were done in the dark. The tissue was incubated with the secondary fluorescent antibody (Alexia555-anti-Rabbit, 1:200) for 1 hour at room temperature in the dark. The second antibody was decanted, and the slides were washed 3 times with TBS for 5 min each time. The slides were incubated with DAPI for 5 min and rinsed with PBS. Coverslips were mounted with a drop of mounting (DAKO) and were sealed with nail polish to prevent drying and movement. The slides were stored in the dark at 4°C and the cells were observed on the confocal microscope Carl Zeiss LSM 700.

### Tumor cell isolation from the lung

To isolate GC cells from metastatic organs, mice received tail vein or footpad injection were sacrificed at indicated time points and their lungs or lymph nodes were taken out, DMEM medium was poured on the surface to keep moist. The tissue was cut into pieces using a scalpel and scissor. Once finally chopped, the minced tissue was transferred to a 15mL tube. 1mL digestion buffer (100μl liberase from Roche in 900uL DMEM) was added and the mixture was kept in 37°C shaker water bath for 1 hours to produce single cell suspensions. After digestion, the cells were centrifuged and resuspended in 5mL trypsin for another 5min. Cells were then resuspended in culture medium and filtered using 70μm cell strainer for running on flow cytometry.

### Statistical analysis

All *in vitro* experiments were performed in triplicate. Data was shown as mean±SD. The survival data was analyzed by Kaplan-Meier method and *P* value was calculated with log-rank analysis. Mann-Whitney test was used to compare the expression level of ARHGAP15 between different groups of human samples. The correlation between ARHGAP15 and RAC1 was analyzed with Spearman correlation (for TMA slides) or Pearson correlation (for transcriptional data from TCGA participants). For in vitro experiments, statistical *P* values were analyzed by a two tailed student *t* test or One-way ANOVA. All statistical analysis was performed by GraphPad 8.0 software. All the tests were two-sided and considered statistically significant if *P*<0.05.

## Supporting information

S1 Fig **(A)**: Comparison of overall survival between GC patients with or without lymph node metastasis with TCGA data. **(B)**: Comparison of overall survival between GC patients with no lymph node metastasis, with less than or more than 30% metastasis involved lymph nodes according to TCGA data.
(TIF)

S2 Fig NUGC4 cells carrying vector or *ARHGAP15* were intravenously injected into the mice and metastatic status was monitored at indicated time points with the help of *in vivo* imaging. (TIF)

S3 Fig **(A)**: Consecutive observations for the bioluminescent signal of intravenously inoculated tumor cells in time series by *in vivo* imaging. **(B)**: The number of flux photons detected from murine lungs at indicated time points was shown on a log scale.
(TIF)

S4 Fig Correlation between ARHGAP15 and RAC1 based on TCGA transcriptional sequencing data (TCGA Firehose Legacy, 415 patients). (TIF)

S5 Fig Representative images show the result of SGC7901 cells with or without overexpression of ARHGAP15 in cell migration (A) and invasion (B). Five random fields of vision were selected to count the migrated/invaded cells for each well and the statistical result of which was shown in the bar chart. Data was shown as mean±SD. ns: no significance.
(TIF)

S1 Uncropped Images The file contains all uncropped images of Western Blot assays. (TIF)
