# Supplementary figures and images for: ARHGAP15 promotes metastatic colonization in gastric cancer by suppressing RAC1-ROS pathway
Source: PLoS Genet. 2023 Feb 21;19(2):e1010640. doi: 10.1371/journal.pgen.1010640 (PMC9983873; doi:10.1371/journal.pgen.1010640)

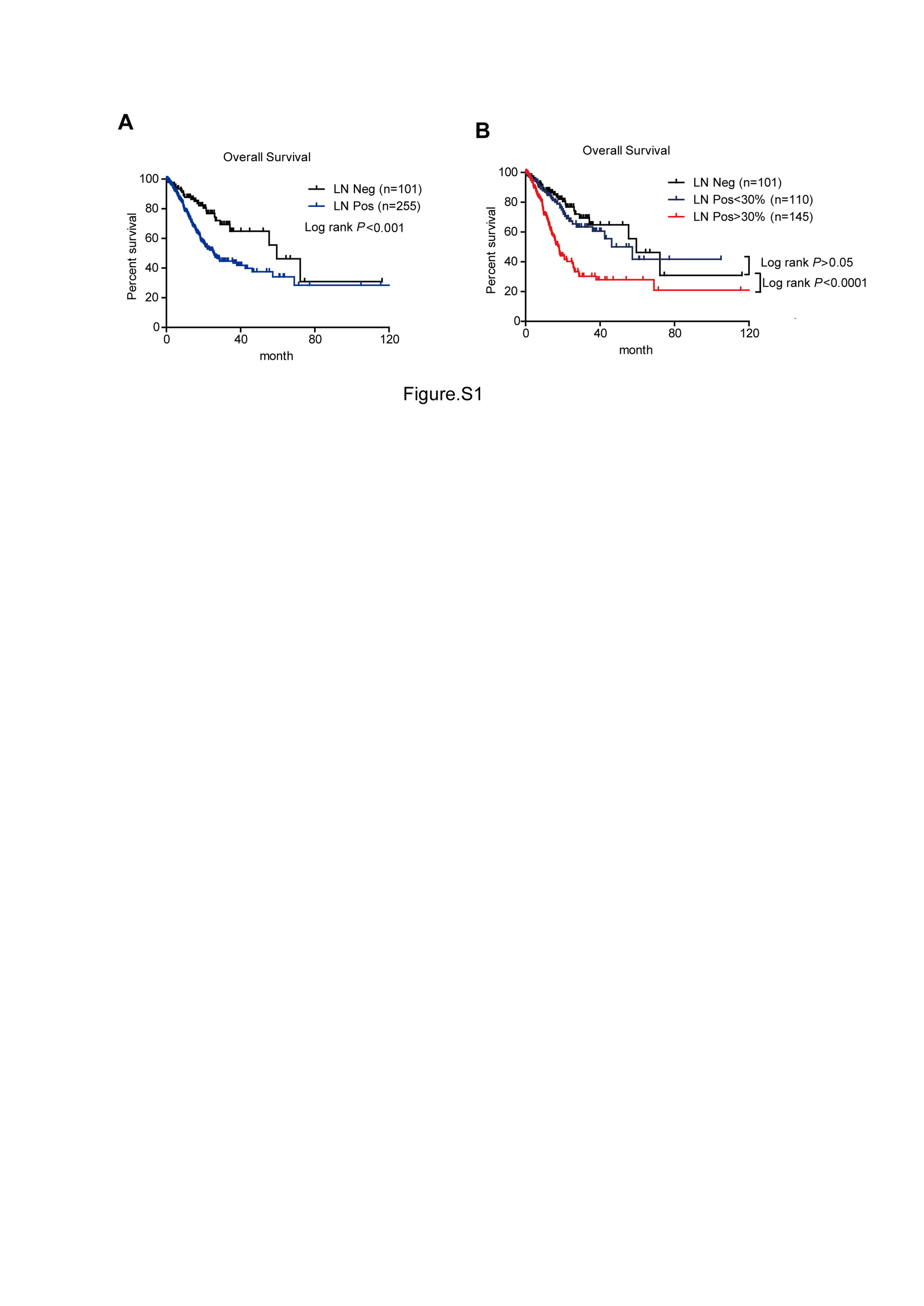

Supplement: S1 Fig — (A): Comparison of overall survival between GC patients with or without lymph node metastasis with TCGA data. (B): Comparison of overall survival between GC patients with no lymph node metastasis, with less than or more than 30% metastasis involved lymph nodes according to TCGA data. (TIF) [file pgen.1010640.s001.tif]

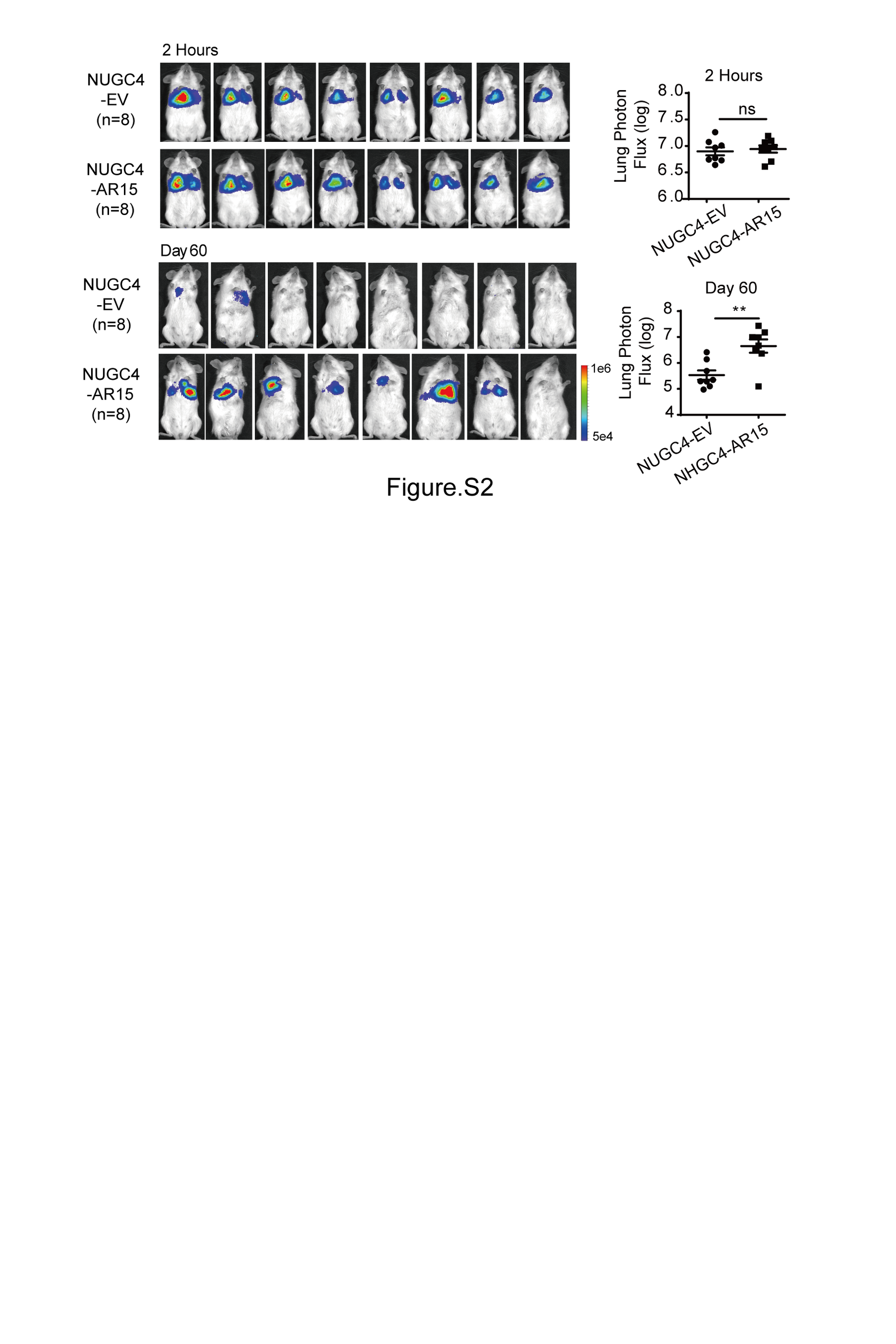

Supplement: S2 Fig — (TIF) [file pgen.1010640.s002.tif]

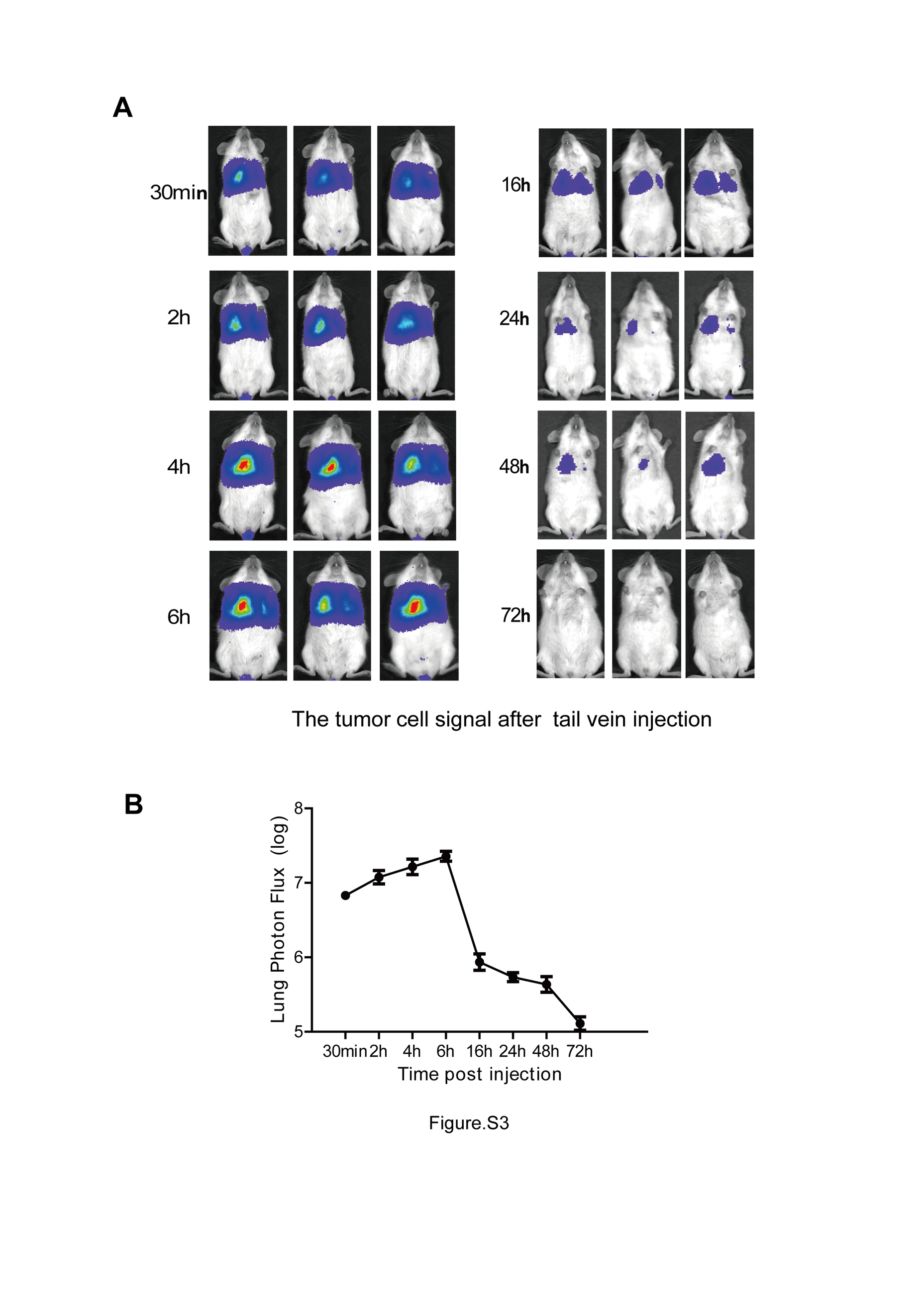

Supplement: S3 Fig — (A): Consecutive observations for the bioluminescent signal of intravenously inoculated tumor cells in time series by in vivo imaging. (B): The number of flux photons detected from murine lungs at indicated time points was shown on a log scale. (TIF) [file pgen.1010640.s003.tif]

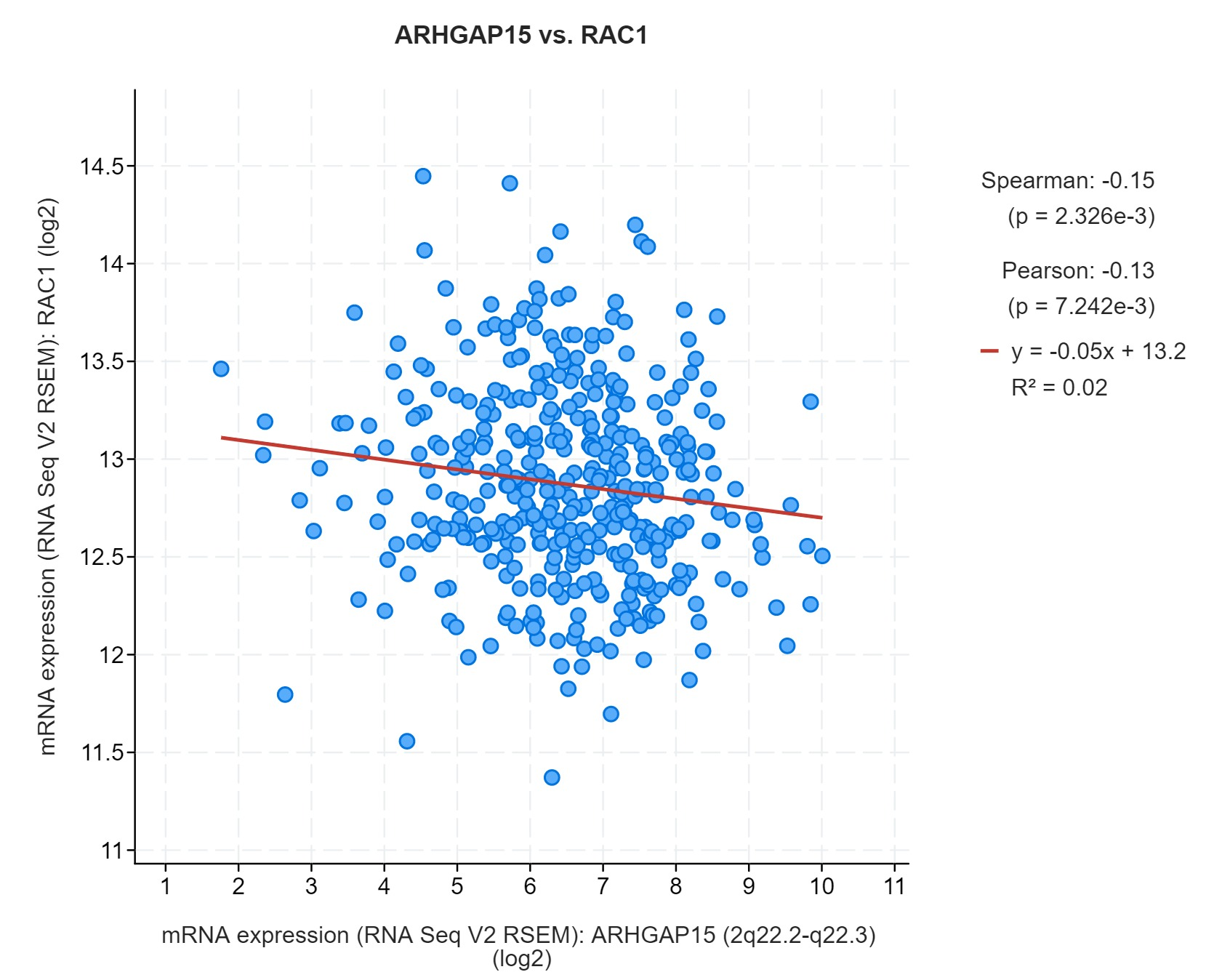

Supplement: S4 Fig — (TIF) [file pgen.1010640.s004.tif]

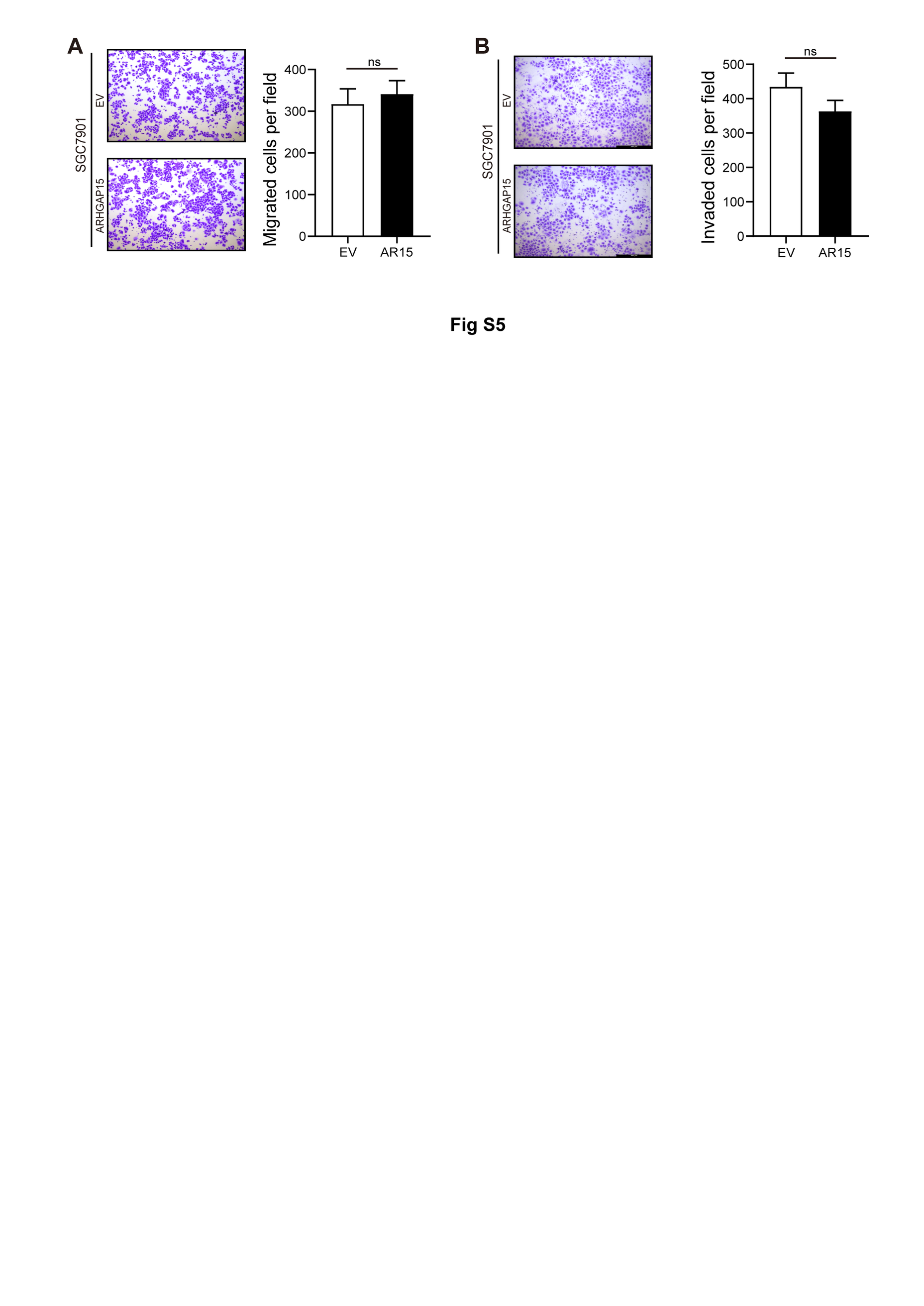

Supplement: S5 Fig — Five random fields of vision were selected to count the migrated/invaded cells for each well and the statistical result of which was shown in the bar chart. Data was shown as mean±SD. ns: no significance. (TIF) [file pgen.1010640.s005.tif]

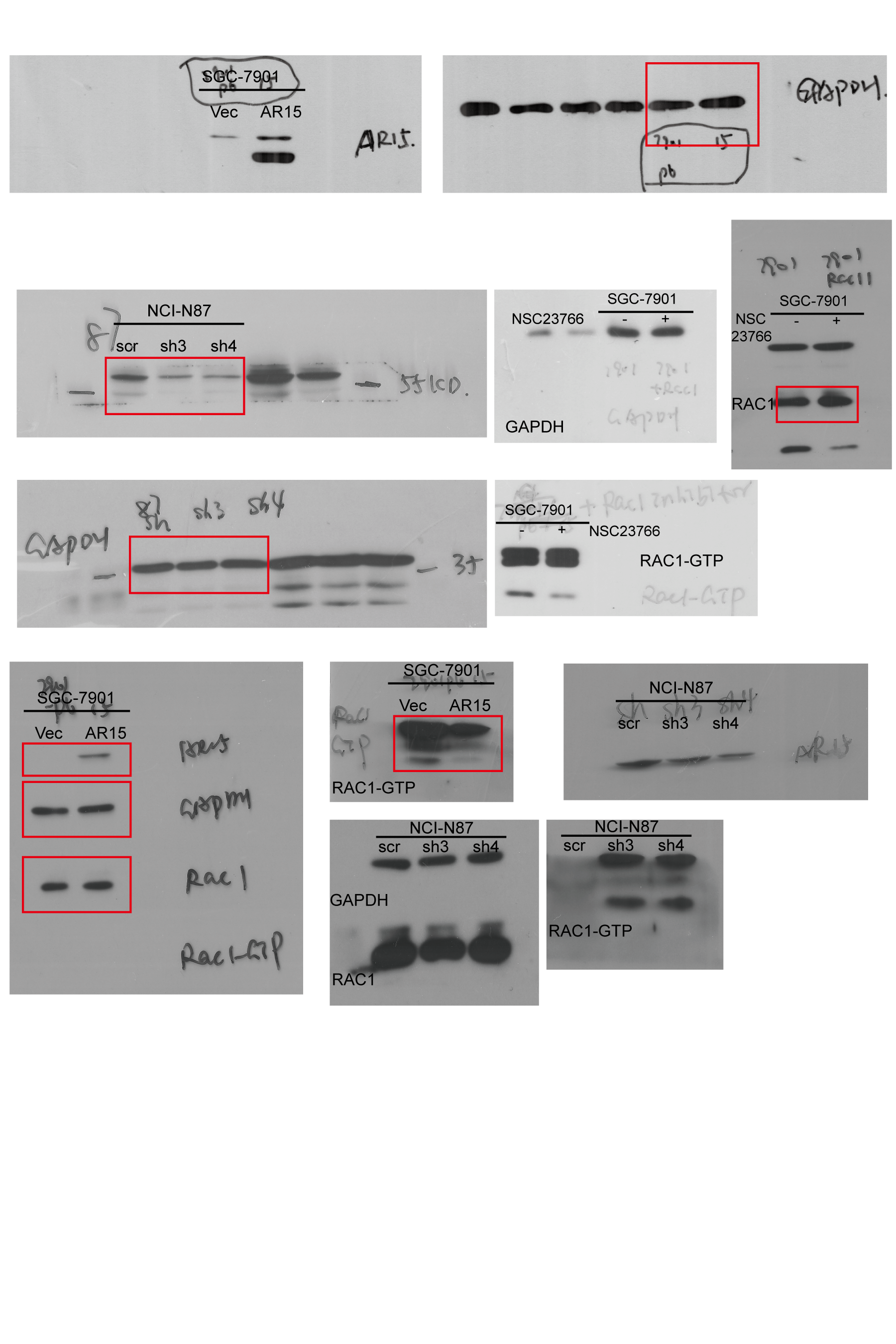

Supplement: S1 Uncropped Images — (TIF) [file pgen.1010640.s006.tif]
